# Appendiceal torsion in Ehlers-Danlos syndrome: A case report of a rare phenomenon in a rare disease

**DOI:** 10.1016/j.ijscr.2020.06.084

**Published:** 2020-06-24

**Authors:** Soobin Kim, Dustin Baldwin, Bernardo Duarte

**Affiliations:** Department of Surgery, Division of General, Minimally Invasive and Robotics, University of Illinois at Chicago, 840 S. Wood Street, Suite 435E, Chicago, IL 60612, United States

**Keywords:** Appendiceal torsion, Appendicitis, Case report, Connective tissue disorders, Ehlers-Danlos syndrome, Volvulus

## Abstract

**Introduction:**

Appendiceal torsion is a rare phenomenon with only a few reported cases and never in a patient with Ehlers-Danlos syndrome (EDS). We present the first case of appendiceal torsion leading to acute appendicitis in a patient with EDS.

**Presentation of case:**

A 36-year-old male with history of musculoskeletal EDS presented with one week of abdominal pain localizing to the right lower quadrant. He was evaluated in the emergency department with a complete set of labs and CT abdomen/pelvis. The CT scan showed acute appendicitis, but did not mention any appendiceal torsion. He was taken to the operating room for a standard laparoscopic appendectomy. Intraoperatively, there was inflammation at the tip of the appendix with a 270-degree torsion of the mid-appendix and mesoappendix. After untwisting, the appendix was safely removed from the base of the cecum. Patient recovered well postoperatively and no masses or tumors were identified on final pathology.

**Discussion:**

Primary appendiceal torsion occurs without an identifiable cause and secondary torsion occurs due to pathology such as carcinoid tumor or other masses. Patients with EDS may be more prone to either torsion as they have hyperelastic skin, joints and ligaments. EDS patients require special workup prior to any operation including cardiopulmonary evaluation and may have unique postoperative complications.

**Conclusion:**

The ligamentous hypermobility of EDS may have predisposed our patient to appendiceal torsion and acute appendicitis. This is a rare phenomenon and may be underdiagnosed in EDS patients. Practitioners should be aware of this manifestation of EDS.

## Highlights

• Appendiceal torsion, a rare phenomenon, may be seen in Ehlers-Danlos syndrome.
• Appendiceal torsion is clinically indistinguishable from acute appendicitis.
• Ehlers-Danlos syndrome contributes to surgical problems due to fragility of tissue.

## 1 Introduction

Appendiceal torsion or volvulus is a rare phenomenon. The first case of appendiceal torsion was reported over 100 years ago in 1918 [1]. Since then, approximately 30 cases have been reported in the literature, involving both children and adults [2]. The pathophysiology of appendiceal torsion involves both primary and secondary forms. Primary appendiceal torsion is a twisting of the mesoappendix causing acute appendiceal ischemia without a secondary cause. Secondary appendiceal torsion is caused by a distinct pathology such as cystadenoma or carcinoid tumor making the appendix susceptible to torsion [2].

Although not as rare as appendiceal torsion, Ehlers-Danlos syndrome (EDS) is an infrequent disease affecting roughly 1 in 5000 people [3]. EDS is a connective tissue disorder that can be manifested in multiple organ systems including skin, joints, blood vessels and abdominal organs. Classically, it is described in patients with hyperflexible joints and hyperextensible skin due to mutations in Type V collagen [4]. Gastrointestinal manifestations include hiatal hernia, rectocele, rectal prolapse and diverticular disease [5].

We present the first case of appendiceal torsion in Ehlers-Danlos syndrome. Although acute appendicitis has been reported in EDS [6], we could not find any reporting of appendiceal torsion. We hope to add to the literature a possible manifestation of EDS, specifically appendiceal torsion. This case was handled at an academic institution and operation was performed by an experienced attending general surgeon. The case report has been presented in accordance with the SCARE criteria [7].

## 2 Presentation of case

Patient is a 36-year-old Caucasian male with past medical history of EDS who presented to the emergency department (ED) of an urban, academic center with diffuse abdominal pain and cramping for nearly one week. Pain was initially epigastric, but then migrated to his right lower quadrant. He endorsed nausea and loose stools but no vomiting, fevers or dysuria. He had never had this type of pain before. Past medical history was significant for EDS which manifested with hypermobility and hyperelasticity of joints and skin with no other known manifestations. To our knowledge, it is unclear how he was diagnosed with EDS, either through genetic testing, clinical symptoms or tissue diagnosis. Regardless, he was diagnosed with EDS prior to arrival and had previously received treatment. Along with EDS, he had a history of gastroesophageal reflux, attention deficit hyperactivity disorder and depression. He denied any past surgical history or any significant family history including EDS. Physical exam revealed hypermobility of joints, no significant cardiopulmonary findings, soft, nondistended abdomen with tenderness to palpation in the right lower quadrant, consistent with acute appendicitis.

On arrival, vitals were within normal limits and a full set of labs and an abdomen/pelvis CT scan with intravenous and oral contrast were obtained. The CT scan showed a dilated appendix (up to 1.4 cm) with mucosal thickening, adjacent fat stranding and edema. There was no mention of appendiceal torsion, masses or lymphadenopathy. There was no fecalith identified. No other non-invasive methods such as abdominal ultrasound were used for diagnosis. Laboratory studies revealed WBC of 7.7, HGB of 15.3, PLT of 298. He was diagnosed with acute appendicitis. He was given IV cefoxitin and made NPO for surgery.

**Fig. 1:**
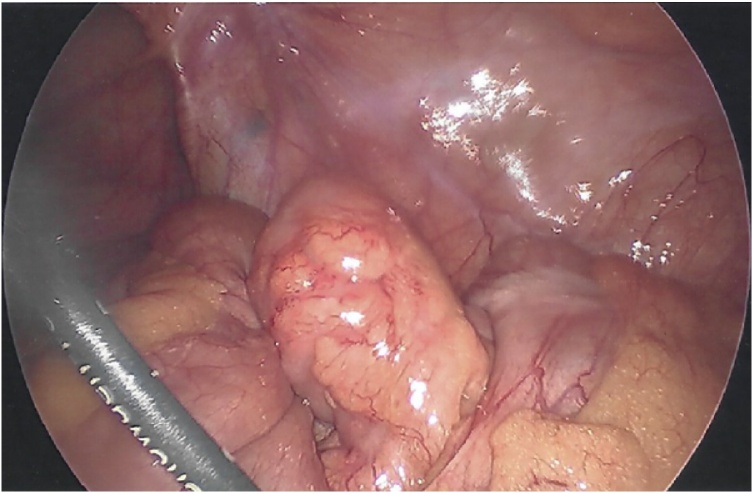
Inflammation at the tip of the appendix.

**Fig. 2:**
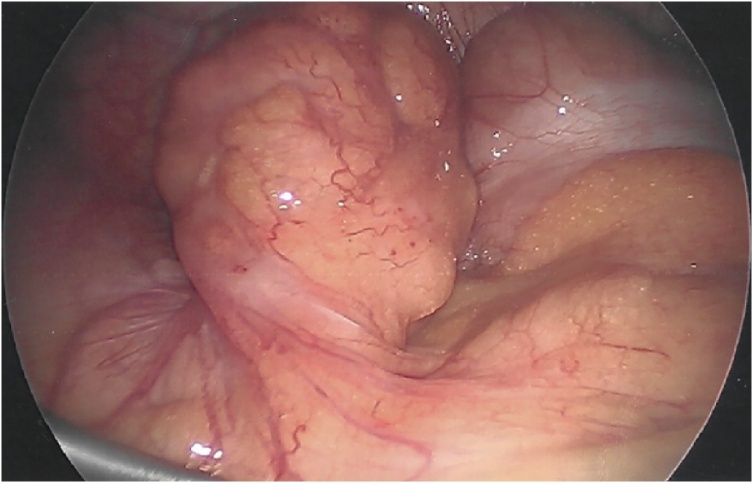
270-degree twisting of the mid-appendix and mesoappendix from medial to anterior position.

**Fig. 3:**
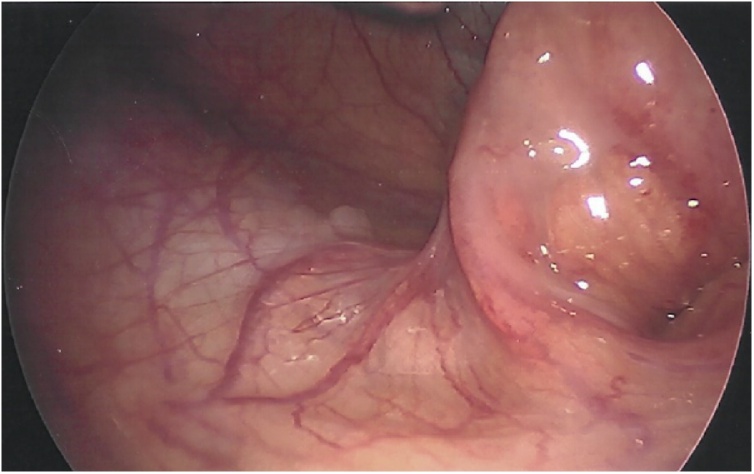
Appendiceal torsion.

He was taken to the operating room for a standard laparoscopic appendectomy. Patient was placed supine and underwent general anesthesia. Foley catheter was placed preoperatively. Open technique at the umbilicus was utilized to gain pneumoperitoneum with a 12 mm port. Next, two 5 mm ports were placed in the left lower quadrant and above the pubic symphysis. To increase our visualization of the appendix, we placed the patient in steep Trendelenburg and rotated right side up. It was at this point the appendix came into view. The distal appendix was swollen with erythema and inflamed mesoappendix (Fig. 1). In the mid-appendix there was 270-degree twisting of the mesentery and appendix, rotating from medial to anterior position (Fig. 2, Fig. 3). We untwisted the appendix, exposing a grossly normal appendix proximal to the torsion. We then carefully dissected out the base of the appendix at the cecum. Using a laparoscopic stapler we divided the appendix off the cecum and divided the mesoappendix. There was no gross spillage of the appendix and no appendiceal masses or lymphadenopathy were identified. The appendix was removed and sent to pathology. We examined the rest of the abdomen and no other abnormalities were noted.

Postoperatively he was discharged the following day after tolerating general diet. Two weeks later, he returned to the clinic with no issues. On the physical exam, the trocar sites were well healed. Pathology on the specimen returned simply with “Acute Appendicitis.” There was no mention of any appendiceal tumors.

## 3 Discussion

Appendiceal torsion is rare and clinically indistinguishable from acute appendicitis. As with our patient, preoperative radiological imaging is generally unrevealing, and this condition is often diagnosed during the operation. While there have been no reported cases of appendiceal torsion in EDS patients, there have been reported cases of stomach and testicular torsion [8,9]. Similar to skin hyperelasticity and joint hyperlaxity, ligamentous laxity seen in EDS may make these patients more prone to torsion of abdominal organs.

In our patient, primary torsion of the appendix may have been the initial event leading to ischemia and ultimately appendicitis. Alternatively, tip appendicitis may have led to inflammation, edema and a subsequent lead point for appendiceal torsion. Traditionally, secondary appendiceal torsion is attributed to a mass (cystadenocarcinoma, carcinoid tumor) causing twisting of the mesentery, not necessarily inflammation. We cannot definitively say what caused the torsion, although we would hedge toward a primary torsion leading to acute appendicitis. Either way, the patient was appropriately managed and underwent a safe laparoscopic appendectomy.

Surgeries in patients with EDS are associated with significant complications, including poor wound healing, dehiscence and intraoperative bleeding [10]. Poor wound healing is a characteristic feature of classical type EDS with poor or absent type V collagen. Type V collagen is a regulatory fibrillar protein that strengthens tissue scaffolds by interaction with other collagens. The absence of this protein contributes to basic surgical problems: extreme fragility of tissue and blood vessels, decreased strength of tissue and ability to hold sutures, and increased bleeding risk [10]. This causes prolonged healing time and wide atrophic scars. Many patients seek the help of facial and plastic surgeons to help amend and close large wounds. Specifically in patients undergoing appendectomy, poor wound healing may lead to higher rates of appendiceal staple line dehiscence which could lead to localized abscess formation, frank peritonitis or post-appendectomy colocutaneous fistula [11].

Preoperative recommendations for the management of classic EDS patients include cardiology assessment with echocardiogram to assess for aortic root dilation and mitral valve prolapse. Blood type and crossmatch testing should be performed prior to surgery in case of uncontrolled bleeding. In addition, the anesthesiology team must be informed of EDS diagnosis due to reported cases of cervical spine and mandibular instability, and skin trauma during intubation [3]. During the operation, careful handling of tissues to avoid bleeding and overstretching is advised. Finally, skin closure should be with well approximated edges and tension free sutures. Ideally, these principles are applied with every operative case; however, in patients with EDS extra attention and time needs to be given to avoid mishaps.

## 4 Conclusion

To our knowledge, this is the first case report in the literature of an appendiceal torsion in a patient with Ehlers-Danlos syndrome. This case serves as evidence of another manifestation of EDS. The ligamentous hypermobility of EDS may have predisposed our patient to appendiceal torsion and acute appendicitis. This is a rare phenomenon and may be underdiagnosed in the EDS patients.

## Declaration of Competing Interest

The authors have no conflicts of interest.

## Funding

There was no source of funding for the research.

## Ethical approval

The Institutional Review Board is not required for publication of a case report at our institution.

## Consent

Written informed consent was obtained from the patient for publication of this case report and accompanying images. A copy of the written consent is available for review by the Editor-in-Chief of this journal on request.

## Author contribution

Study Concept and Design: Dustin Baldwin, Bernardo Duarte.

Data Collection: Dustin Baldwin. Bernardo Duarte.

Writing of Paper: Soobin Kim, Dustin Baldwin, Bernardo Duarte.

## Guarantor

Bernardo Duarte.

## Provenance and peer review

Not commissioned, externally peer reviewed.
